# Transcriptional response of transposable elements to thermal stress in the Antarctic fish *Trematomus bernacchii*

**DOI:** 10.1038/s41598-025-33487-5

**Published:** 2026-01-07

**Authors:** Edith Tittarelli, Elisa Carotti, Claudia Palladinelli, Marco Barucca, Federica Carducci, Gianfranco Santovito, Elisabetta Piva, Adriana Canapa, Maria Assunta Biscotti

**Affiliations:** 1 Dipartimento di Scienze della Vita e dell’Ambiente , Università Politecnica delle Marche , Via Brecce Bianche, 60131 Ancona, Italy; 2 Scuola Universitaria Superiore Pavia - IUSS, Piazza della Vittoria n.15, 27100 Pavia, Italy; 3 Dipartimento di Biologia, Università di Padova, Via Ugo Bassi 58B, 35121 Padova, Italy

**Keywords:** Ecology, Ecology, Evolution, Genetics, Zoology

## Abstract

Global change and the associated increase in temperature raise serious concerns for the conservation of Antarctic marine biodiversity, which is particularly vulnerable due to the stenothermal nature and highly specialized adaptations of its fauna. *Trematomus bernacchii* (commonly named emerald rockcod), a Southern Ocean-endemic benthic fish, serves as a valuable model organism for investigating the molecular and physiological impacts of climate change in polar ecosystems. Transposable elements (TEs) are of particular interest, as they are known to become activated under stress and to influence genome plasticity and gene regulation. In this study, we examined the transcriptional response of TEs and their silencing mechanisms in the gills and liver of *T. bernacchii* specimens exposed to thermal stress (+ 1 °C and + 3 °C compared to a 0 °C control) for 5 and 15 days. Our results showed that temperature increase triggered a transient activation of TEs, followed by the upregulation of silencing-related genes, including members of the Argonaute family, heterochromatin-associated factors, and components of the NuRD complex. Tissue-specific patterns were observed: the liver exhibited a rapid balance between TE activation and silencing, indicating a coordinated and resilient response, while the gills showed a sustained upregulation of both TEs and silencing genes, likely due to their greater sensitivity to environmental changes. These findings highlighted a complex, dynamic interplay between TEs and their regulatory systems under heat stress, offering new insights into early adaptive responses and potential resilience mechanisms in a cold-adapted species facing climate-induced biodiversity loss.

**Supplementary Information:**

The online version contains supplementary material available at 10.1038/s41598-025-33487-5.

## Introduction

Rates of current global warming have largely exceeded those experienced in the past^1–4^ and changes in seawater temperature are expected to occur on a faster time-scale and with a greater magnitude in the Southern Ocean compared to temperate regions. Indeed, the Southern Ocean is one of the hotspots for global warming^5,6^. Moreover, in Antarctica, the biological cycles of marine organisms are strongly shaped by seasonal abiotic variation, and therefore polar ecosystems could experience a negative impact under the environmental perturbations induced by climate change^7,8^.

Antarctic fish represent a major component of the biomass in the Southern Ocean, and, in an ecological setting, they are a link between low trophic levels and top predators^9,10^. Nototheniidae is one of the five Antarctic families belonging to the suborder Notothenioidei^11^ that is mostly composed of endemic species. They inhabit coastal ecosystems around Antarctica in waters characterized by temperature reaching − 1.9 °C due to the isolation created by the water masses of the Antarctic convergence surrounding the continent^12,13^. Indeed, the Antarctic continental shelf represents one of the most oceanographically constant environments of the Earth. Antarctic fish have acquired a wide range of adaptations to the cold^12–15^, including limited thermal plasticity^16,17^, enabling survival within a restricted range of temperatures. During 10–14 million years of evolution, the thermal stability of the Antarctic environment has driven the loss of coding genes and regulatory networks required for coping with environmental variability^18,19^, reducing the adaptive potential of living species^20^.

In the field of polar biology, *Trematomus bernacchii* is considered a target species for studying the physiological and biochemical response to environmental changes. This species has a ubiquitous distribution around the Antarctic continent and shows a remarkable abundance^21^. *T. bernacchii* has traditionally been described as stenothermic; however, several studies have indicated that this species retains the capacity for thermal acclimation. Davis et al.^16^ have demonstrated metabolic compensation to warming, suggesting that *T. bernacchii* can adjust its metabolism under elevated temperatures. Similarly, Enzor and Place^17^ have reported that oxidative damage and standard metabolic rate exhibit acclimation under warming over time. These findings have indicated that *T. bernacchii* is not strictly stenothermic and can exhibit thermal plasticity. The acclimation capacity of *T. bernacchii* has been the subject of many studies^22–30^. In particular, through transcriptomic studies, functional investigations were extended to the layer of gene expression to better understand how the physiological plasticity influences the response of organisms in the face of global climate change. Several papers have reported the inability of *T. bernacchii* and other notothenioid fishes to mount a heat shock response since no genes encoding heat shock proteins (HSP) (except for *Hsp40*) were upregulated following thermal exposure^31–37^. Nonetheless, microarray analyses have revealed that hundreds of genes related to cellular stress response were differently expressed during recovery from heat exposure, indicating that this kind of reaction to thermal stress was retained in extremely cold-adapted fish^31^. Using a high-throughput RNA-sequencing approach, Huth and Place^32^ have further confirmed the general inability of emerald rockcod to mount a heat shock response in liver, brain and gills even if *Hsp70* showed a major responsiveness in this latter tissue. In 2016, the same authors^33^ have reported that a multi-stressor condition induces a strong initial response followed by a return to near basal levels of expression at longer acclimation times. However, a number of key genes remained up-regulated indicating that *T. bernacchii* has not fully compensated. Although this species could have the physiological plasticity to cope with similar environmental conditions due to climate change, the long-term impacts on populations could reduce growth and reproduction. Recently, the work by Greco and colleagues^37^ has highlighted in *T. bernacchii* an increased responsiveness of the brain compared to gills and muscle and an unexpected downregulation of HSPs in neural tissue. These papers have investigated the transcriptomic responses based on genetic pathways evolved by *T. bernacchii* in response to abiotic variables, particularly thermal stress.

A growing body of literature has underscored the transcriptional responsiveness of transposable elements (TEs) in relation to environmental variability in fish^38–43^. TEs are repetitive sequences that move throughout the genome using a transposition mechanism. TEs can be divided in class I or retroelements if they use an RNA molecule as intermediate during transposition and class II or DNA transposons if they use a DNA molecule for this purpose^44^. The TE transcriptional activity might be associated with the up- or downregulation of nearby genes through regulatory sequences embedded within TEs or TE-derived noncoding RNAs^45–47^. TEs are domesticated by the host genome to rewire gene expression networks allowing the physiological response of organisms, permitting species adaptation and resilience to cope with the effects of climate change. Although most of the consequences of transposition are neutral^48^, others can cause genome instability^49^. Therefore, organisms evolved silencing mechanisms such as those based on the involvement of small RNAs and proteins of the Argonaute superfamily or the KRAB zinc finger proteins and the nucleosome remodeling deacetylase complex (NuRD). These controlling machineries act modulating the heterochromatin status of TE sequences depositing epigenetic marks at DNA and histone levels^50^.

In this study, we investigated the transcriptional response of TEs and their associated silencing mechanisms in the gills and liver of *T. bernacchii* exposed to + 1 and + 3 °C with respect to the control temperature (0 °C). The fish gills represent the body part in direct contact with external environment and thus are expected to be sensitive to temperature changes^51^; the liver hosts a wide variety of pathways included those related to thermoregulation^52^. Thermal stress induced a transient activation of TEs, followed by the upregulation of silencing mechanisms aimed at preserving genomic stability in *T. bernacchii*. Notably, the two tissues displayed distinct responses: the liver showed a more coordinated and resilient reaction, whereas the gills displayed sustained upregulation of both TEs and silencing genes throughout the exposure, likely due to their increased sensitivity to temperature fluctuations.

## Results

### Transposable element composition and sequence divergence in *T. bernacchii *genome and transcriptomes

The scaffold-level genome assembly of *T. bernacchii* spans a total of 867.1 Mb. RepeatMasker analysis indicated that transposable elements (TEs) account for 44.02% of the genome, with DNA transposons representing the most prevalent class (21.73%), followed by LINE retrotransposons (9.77%), LTR retrotransposons (3.83%), and SINE retrotransposons (0.37%) (Supplementary Table S1). The TE landscape, based on Kimura distance analysis, exhibited a prominent peak at low K-values, with the majority of TE copies concentrated below a K-value of 25 (Supplementary Fig. S1). The Kimura two-parameter model was used to estimate nucleotide substitutions (transitions and transversions) between each TE copy and its consensus sequence. Low K-values therefore indicate low sequence divergence and are interpreted as evidence of recent insertions.

Transcribed TEs constituted a smaller proportion of the assembled transcriptomes compared to the genomic content. In both liver and gill tissues, the Kimura distance landscapes displayed comparable patterns, characterized by a sharp peak near K = 0 and a secondary elevation at K-values ranging from 20 to 25 (Supplementary Fig. S2).

### Transcriptional activity of TEs in *T. bernacchii* liver and gills

**Fig. 1:**
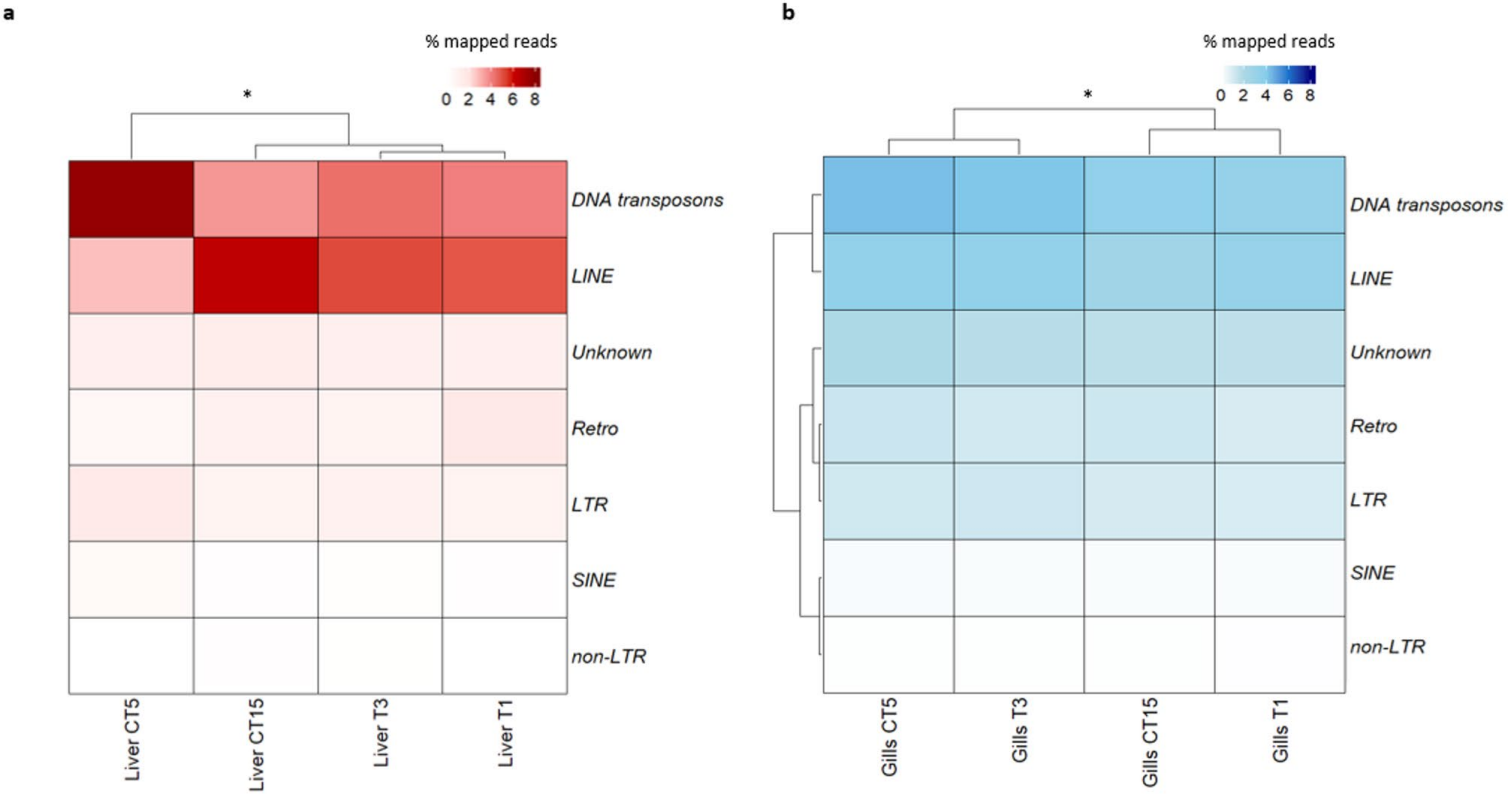
Heatmaps of TEs in liver and gill tissues for each experimental and control groups. (**a**) Liver panel, transcriptional contribution of TEs activity as percentage of mapped reads is reported for the analyzed conditions. (**b**) Gills panel, transcriptional contribution of TEs activity as percentage of mapped reads is reported for the analyzed conditions. “Unknown” means TEs that are not specifically classified as DNA transposons, LINE, SINE, and LTR retrotransposons; “non-LTR” retrotransposons are referred to retrotransposons that are not specifically classified as LINE or SINE retrotransposons; “Retro” is referred to retrotransposons that are not specifically classified as LINE, SINE, LTR or non-LTR retrotransposons. Statistically significant differences are presented as * for *p* < 0.05, ** for *p* < 0.01, and *** for *p* < 0.001.

Specimens of emerald rockcod were exposed to temperatures of + 1 °C (T1) and + 3 °C (T3) to simulate future scenarios due to global warming and compared with corresponding control groups sampled after 5 (CT5) and 15 (CT15) days. At each time point, the transcriptional activity of TEs was assessed in liver and gill tissues (Fig. 1). Overall, a substantial transcriptional contribution from DNA transposons and LINE retrotransposons was observed, followed by LTR retrotransposons. In contrast, SINE retrotransposons exhibited generally low expression levels in both tissues under all experimental conditions, exception for the control liver sample at day 5, which showed comparatively higher expression (Fig. 1a). Heatmap analyses revealed distinct TE transcriptional profiles between control and temperature-exposed samples at both time points (5 and 15 days) in both tissues (Fig. 1). Notably, in the liver, TE expression pattern of CT5 was significantly distinct from all other samples including the two exposed groups (Fig. 1a). In the gills, the transcriptional profiles under + 1 °C and + 3 °C were significantly different, but T1 was not significantly different from CT15 and T3 was not significantly different from CT5. CT5 and CT15 were significantly different from each other (Fig. 1b).

**Fig. 2:**
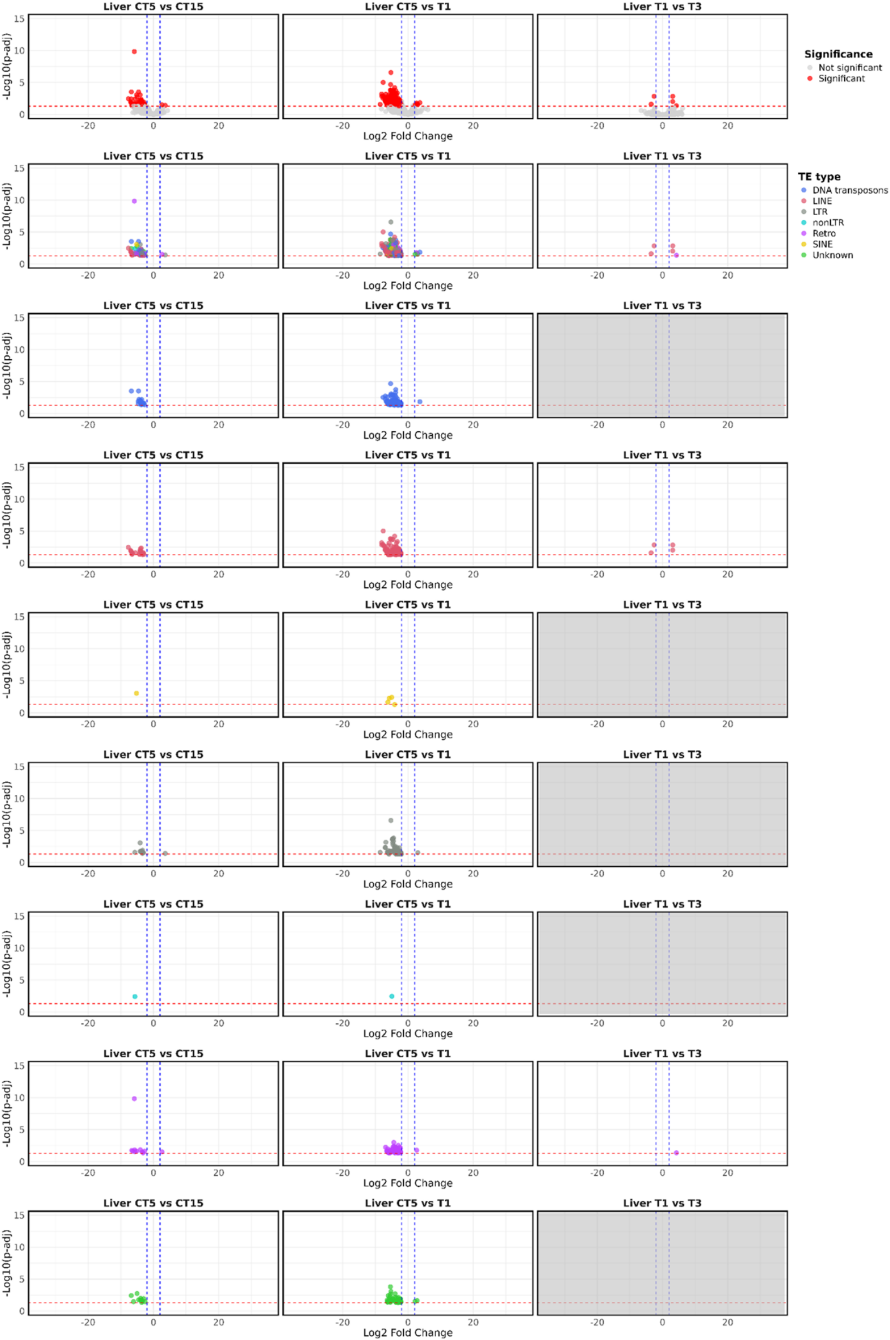
Volcano plot of differentially expressed TEs (DETEs) between test vs. control for liver samples. The top row shows significant (red) and non-significant (light grey) TEs. In the second row TEs are represented as color-codes by typology (DNA transposons in blue, LINE retrotransposons in coral, LTR retrotransposons in grey, SINE retrotransposons in yellow, Retro in purple, non-LTR retrotransposons in cyan, and unknown in green). Subsequent rows are related to single TE classes. “Unknown” means TEs that are not specifically classified as DNA transposons, LINE, SINE, and LTR retrotransposons; “non-LTR” retrotransposons are referred to retrotransposons that are not specifically classified as LINE or SINE retrotransposons; “Retro” is referred to retrotransposons that are not specifically classified as LINE, SINE, LTR or non-LTR retrotransposons. The blue dashed lines indicate the significant thresholds for Log2 Fold Change >|2|, while the red dashed the statistically significant threshold (p-adj < = 0.05). Light grey plots indicate comparisons for which no DETEs were identified.

In liver, differential expression analysis allowed the identification of 365 differentially expressed transposable elements (DETEs) in the T1 vs. CT5 comparison, of which 360 were downregulated in the T1 condition. Among these, 124 were LINE retrotransposons, 86 DNA transposons, 64 L retrotransposons, 43 unknown elements, 38 retrotransposons, four SINE retrotransposons, and one non-LTR retrotransposon (Supplementary Table S2). No DETEs were detected in the comparison between CT15 and T3 (Fig. 2).

**Fig. 3:**
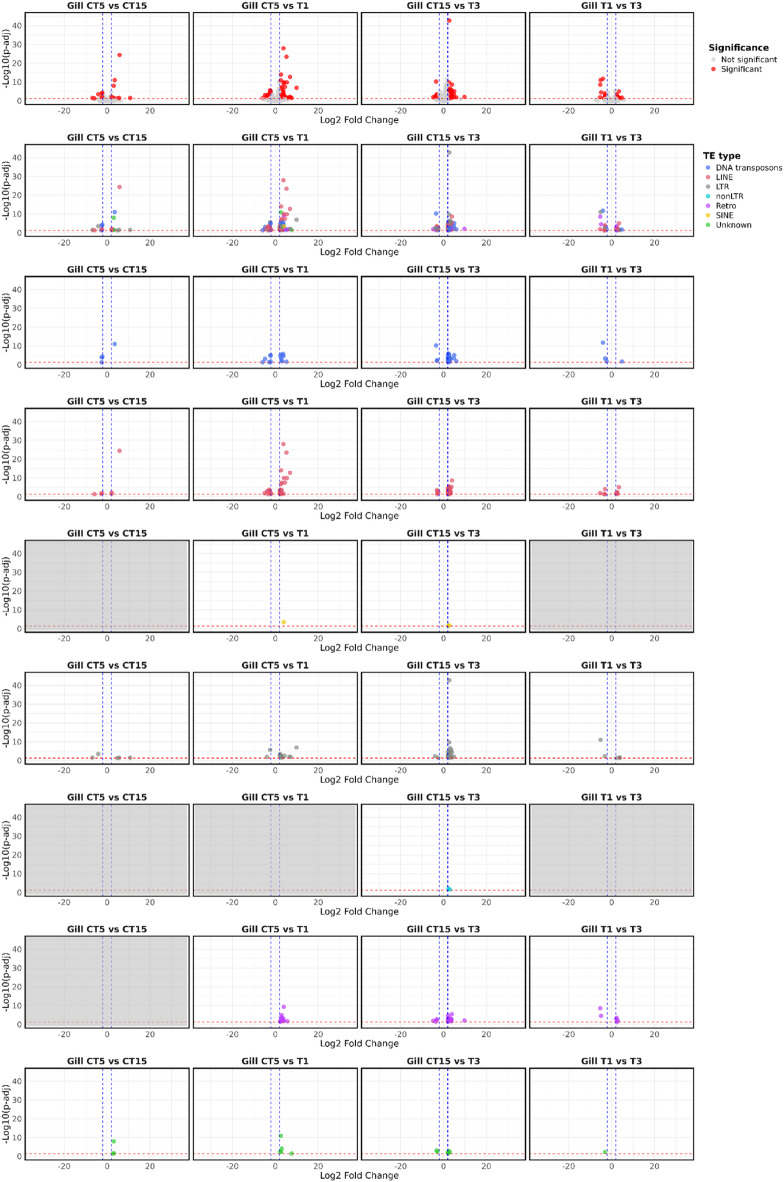
Volcano plot of differentially expressed TEs (DETEs) between test vs. control for gills samples. The top row shows significant (red) and non-significant (light grey) TEs. In the second row TEs are represented as color-codes by typology (DNA transposons in blue, LINE retrotransposons in coral, LTR retrotransposons in grey, SINE retrotransposons in yellow, Retro in purple, non-LTR retrotransposons in cyan, and unknown in green). Subsequent rows are related to single TE classes. “Unknown” means TEs that are not specifically classified as DNA transposons, LINE, SINE, and LTR retrotransposons; “non-LTR” retrotransposons are referred to retrotransposons that are not specifically classified as LINE or SINE retrotransposons; “Retro” is referred to retrotransposons that are not specifically classified as LINE, SINE, LTR or non-LTR retrotransposons. The blue dashed lines indicate the significant thresholds for Log2 Fold Change >|2|, while the red dashed the statistically significant threshold (p-adj < = 0.05). Light grey plots indicate comparisons for which no DETEs were identified.

In gills, fewer DETEs were identified compared with liver, with most elements showing upregulation (Fig. 3). In the T1 vs. CT5 comparison, 86 DETEs were detected, including 68 upregulated and 18 downregulated elements (Supplementary Table S2). Among the upregulated TEs, 26 were LINE retrotransposons, 15 L retrotransposons, eight DNA transposons, 13 retrotransposons, five unknown elements, and one SINE retrotransposon. The downregulated group included ten LINE retrotransposons, six DNA transposons, and two LTR retrotransposons. The T3 vs. CT15 comparison yielded 138 DETEs, with 122 upregulated and 16 downregulated. Within the upregulated set, 48 were LINE retrotransposons, 24 L retrotransposons, 23 DNA transposons, 16 retrotransposons, seven unclassified, two SINE retrotransposons, and two non-LTR retrotransposons. Among the downregulated elements, LINE retrotransposons (5 elements) were again the most represented, followed by retrotransposons (4 elements), DNA transposons (3 elements), LTR retrotransposons (2 elements), and unknown elements (2 elements) (Supplementary Table S2). Comparisons within each tissue between control time points (CT15 vs. CT5) and between thermal exposures (T3 vs. T1) revealed a limited number of DETEs (Supplementary Table S2).

**Fig. 4:**
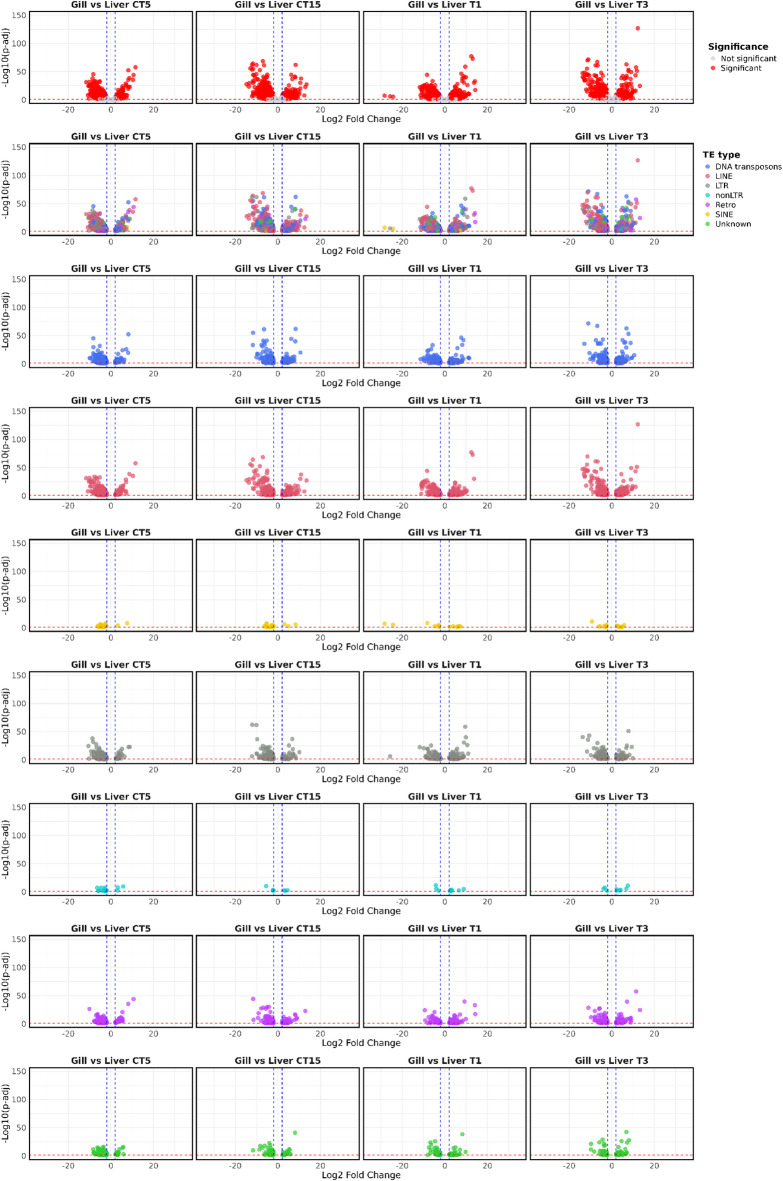
Volcano plot of differentially expressed TEs (DETEs) between test vs. control for comparisons between liver and gills samples. The top row shows significant (red) and non-significant (light grey) TEs. In the second row TEs are represented as color-codes by typology (DNA transposons in blue, LINE retrotransposons in coral, LTR retrotransposons in grey, SINE retrotransposons in yellow, Retro in purple, non-LTR retrotransposons in cyan, and unknown in green). Subsequent rows are related to single TE classes. “Unknown” means TEs that are not specifically classified as DNA transposons, LINE, SINE, and LTR retrotransposons; “non-LTR” retrotransposons are referred to retrotransposons that are not specifically classified as LINE or SINE retrotransposons; “Retro” is referred to retrotransposons that are not specifically classified as LINE, SINE, LTR or non-LTR retrotransposons. The blue dashed lines indicate the significant thresholds for Log2 Fold Change >|2|, while the red dashed the statistically significant threshold (p-adj < = 0.05).

When comparing liver and gills, a substantial number of DETEs was observed, primarily involving LINE retrotransposons, followed by DNA transposons and LTR retrotransposons (Fig. 4, Supplementary Table S2). Downregulated DETEs were predominant in comparisons between control conditions (CT5 and CT15), whereas comparisons between temperature treatments showed a more balanced distribution between up- and downregulated elements.

### Transcriptional profiles of genes involved in TE silencing

To better understand the regulation of TEs under thermal stress, we analyzed the expression profiles of key genes implicated in TE silencing pathways. These included components of the RNA interference machinery (e.g., *Argonaute* subfamily genes), genes encoding proteins involved in heterochromatin formation, and genes encoding epigenetic regulators such as proteins of the NuRD complex.

**Fig. 5:**
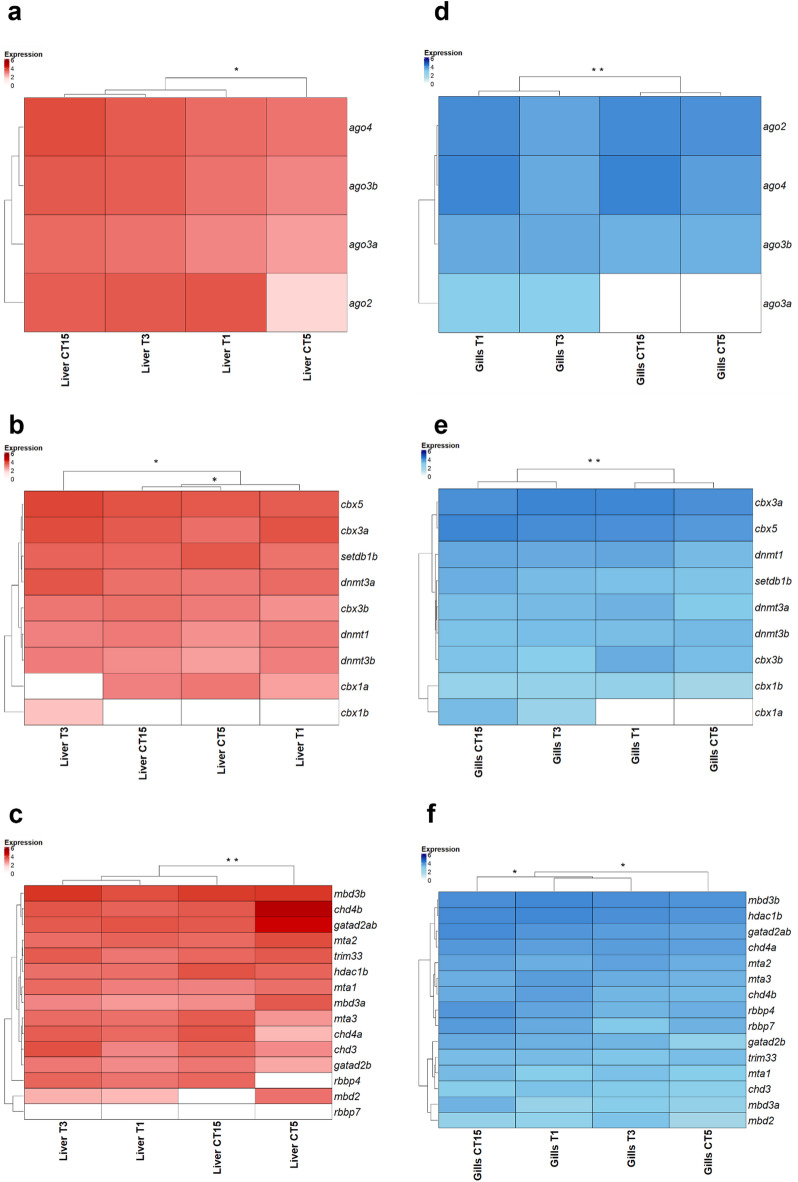
Heatmaps of expression profiles of genes encoding proteins involved in TE silencing in liver and gill tissues. (**a**) Heatmap based on logarithmic TPM values of Argonaute genes at four experimental conditions in liver. (**b**) Heatmap based on logarithmic TPM values of genes involved in heterochromatinization at four experimental conditions in liver. (**c**) Heatmap based on logarithmic TPM values of genes involved in NuRD complex at four experimental conditions in liver. (**d**) Heatmap based on logarithmic TPM values of Argonaute genes at four experimental conditions in gills. (**e**) Heatmap based on logarithmic TPM values of genes involved in heterochromatinization at four experimental conditions in gills. (f) Heatmap based on logarithmic TPM values of genes involved in NuRD complex at four experimental conditions in gills. Statistically significant differences are presented as * for *p* < 0.05, ** for *p* < 0.01, and *** for *p* < 0.001.

In the liver, no transcripts corresponding to *Ago1* were detected under any conditions. However, transcripts for *Ago2*, *Ago3a*, *Ago3b*, and *Ago4* were expressed in both control and test samples. Notably, all four genes exhibited increased expression in the T1 group compared with CT5, whereas expression patterns between CT15 and T3 remained largely comparable (Fig. 5a).

Regarding genes associated with heterochromatin formation, expression profiles of control samples (CT5 and CT15) were more closely related than those of exposed groups. Differential expression was observed in both T1 vs. CT5 and T3 vs. CT15 comparisons, with the latter showing more pronounced changes. In particular, an upregulation of *cbx1b*,* cbx5*, *cbx3a*, *setdb1b*, *dnmt3a*, and *dnmt3b* was recorded in T3 relative to CT15 (Fig. 5b). Genes encoding components of the NuRD complex also displayed altered expression between CT5s and T1, whereas no significant differences were detected between CT15 and T3. However, the two thermally stressed groups (T1 and T3) showed broadly similar transcriptional profiles (Fig. 5c).

In the gills, *Ago1* transcripts were absent under all conditions. The other *Argonaute* genes (*Ago2*, *Ago3a*, *Ago3b*, and *Ago4*) showed differential expression between controls and exposed samples. In the T1 vs. CT5 comparison, all four genes displayed increased transcriptional activity. Moreover, expression patterns of *Ago* genes were more similar within control groups and within exposed groups than between them (Fig. 5d).

Conversely, genes involved in heterochromatin formation showed similar expression trends between each control and its corresponding exposed group. With the exception of *dnmt3b*, all analyzed genes were upregulated in T1 relative to CT5 (Fig. 5e).

For genes encoding components of the NuRD complex, expression profiles differed between control and exposed samples, with more marked differences observed between CT5 and T1. Additionally, expression profiles were more similar between the two exposed groups than control conditions (Fig. 5f).

## Discussion

Global warming poses a serious threat to Antarctic marine fauna due to the stenothermal nature of the environment and the highly specialized adaptations of its species to cold, stable temperatures. In this context, *T. bernacchii*, a key benthic fish species endemic of the Southern Ocean, represents an important model organism. Its limited thermal tolerance and evolutionary adaptations to the extreme conditions of the Southern Ocean make it particularly valuable for studying the physiological and ecological consequences of climate change in polar ecosystems. Although numerous studies have investigated the susceptibility of the emerald rockcod to the effects of global warming by analyzing genetic pathways^30–37^, the role of TEs has received comparatively little attention and remains underexplored in this species. This study firstly explores TE expression and related silencing gene responses to thermal stress in an Antarctic fish. TEs are key drivers of genome evolution, capable of influencing gene expression, generating genetic diversity, and facilitating rapid adaptation^53–56^. Their activity may be particularly relevant under environmental stress, making them key players in the evolutionary responses of species to climate change^53–58^.

TEs constitute a substantial portion of the *T. bernacchii* genome, with DNA transposons and LINE retrotransposons being the most represented classes. Moreover, the TE sequence divergence analysis showed a peak at low K values, indicating a recent burst of amplification. Most TE copies were distributed below a K-value of 25, indicating the presence of recently inserted elements. These findings were consistent with observations in other teleost species, including those from non-Antarctic environments, in which DNA transposons and LINE retrotransposons are also the most abundant TE classes and a high proportion of recent insertions is commonly observed^59–63^.

It is known that organisms exposed to stress conditions may exhibit TE activation and such mobilization can generate genetic variability that provides the raw material for adaptive evolution^57,64^. Transcriptomic analyses performed in this study revealed active TE expression in the liver and gills of *T. bernacchii* specimens exposed to thermal stress. In the early phase (day 5), in both tissues, heat exposure induced distinct TE expression profiles compared with control conditions, suggesting a rapid transcriptional response to environmental perturbation. This response was more pronounced in the liver, where a greater number of DETEs was observed than in the gills. These findings highlighted that the two tissues have a distinct response in relation to thermal stress as further supported by the high number of DETEs identified when comparing liver and gills. Interestingly, most DETEs in hepatic tissue were downregulated, whereas those in the gills were predominantly upregulated. Indeed, TE activation is not a generalized process but under stress TE repression may also occur^39,64–66^. It has been proposed that stress often triggers repression of more TEs that it activates, serving as a stabilizing mechanism that balances the potential for genetic innovation with the need to limit genome instability caused by TE activation by stress^66^. Our analyses revealed that the majority of DETEs belonged to LINE retrotransposons in both tissues. This suggests that LINE retrotransposons were responsive to heat stress in *T. bernacchii*, indicating a bias towards these elements. Similar associations between specific TE types and stress conditions have been reported in both animal and plant species^67–72^.

A transcriptional response was still detectable in the gills when comparing CT15 and T3 conditions, whereas no DETEs were identified in the liver for the same comparison. The transient yet coordinated modulation of TE expression observed, characterized by early activation followed by later silencing, may reflect a more generalized transcriptional strategy in *T. bernacchii* in response to thermal stress. This pattern has previously been noted for several canonical gene pathways related to cellular stress and plasticity^24,73^, and it is further supported by preliminary transcriptomic data showing similar dynamics in immune- and stress-related genes.

Expression of genes involved in TE silencing, particularly Argonaute proteins, chromatin modifiers, and members of the NuRD complex, supported the hypothesis of an active repression mechanism in response to TE mobilization. In both tissues, *ago2*, *ago3a*, *ago3b*, and *ago4* were upregulated under stress, particularly at T1. This early transcriptional activation aligns with the observed TE expression profiles and may reflect an immediate counter-regulatory response to TE mobilization. At day 15, expression levels stabilized, especially in the liver, suggesting the establishment of a new regulatory balance after initial perturbation.

Genes involved in heterochromatin formation, such as *cbx3a*, *cbx5*, *setdb1b*, *dnmt3a*, and *dnmt3b*, also exhibited significant upregulation, particularly in the liver at T3, indicating reinforcement of transcriptional repression mechanisms at later time points. These epigenetic regulators likely contribute to restoring genome stability following TE activation. The NuRD complex, a key chromatin remodeling factor, showed differential expression in response to thermal stress. The similarity in expression patterns between T1 and T3 samples in both tissues suggested that, once activated, this regulatory complex remains consistently involved in chromatin reorganization under prolonged stress exposure.

Overall, liver and gills exhibited distinct TE responses to thermal stress, emphasizing the importance of tissue-specific regulation in stress adaptation. Although both tissues showed early transcriptional activation at T1, in T3 the liver no longer displayed any differentially expressed TEs compared with its control, suggesting a recovery in hepatic tissue that was not observed in the gills. The liver showed a more coordinated and possibly repressive response to TE activation, the gills exhibited sustained upregulation of TEs and silencing genes, potentially reflecting their direct interface with the external environment and greater sensitivity to temperature fluctuations^33^.

With due caution, we are confident that the observed results were minimally influenced by housing conditions during the acclimation period. However, it must be acknowledged that the lack of feeding may have influenced the results. Antarctic fish have exceptionally low metabolic rates compared to temperate and tropical species^74^, and although they are known to feed infrequently during summer and may completely cease feeding during winter, we cannot rule out hunger may still influence specific physiological processes. Stepanowska and Nędzarek^75^ have reported that up to 50 days of starvation periods caused no significant changes in body weight or chemical composition in two Antarctic species, *Notothenia coriiceps* and *N. rossii*. The same authors reported a marked reduction in excretion rates in starved fish, indicating changes in metabolic activity after the first day of starvation, with very limited changes thereafter. This suggests that the fasting period used in our experiment may contribute to some of the temporal differences observed, particularly in the initial phase. However, ensuring frequent nutrition can also increase metabolism at feeding time, temporarily increasing oxygen consumption. It is known that this factor increases the production of reactive oxygen species, leading to the activation of the antioxidant system, which in these fish is very efficient^76^, and to post-transcriptional regulation^77^ that may affect the results.

## Conclusions

Together, these results suggested that thermal stress in *T. bernacchii* triggers transient TE activation, followed by upregulation of silencing pathways to restore genomic stability. The observed transcriptional patterns underscore the responsiveness of both the RNA interference machinery and epigenetic regulators to environmental stress, highlighting their critical role in genome defense. The tissue-specific responses further indicated differential regulatory strategies that may be vital for maintaining physiological function under changing environmental conditions. This study sheds light on an underexplored aspect of Antarctic fish biology, suggesting that even in highly cold-adapted fish, TEs and their regulators may contribute to genomic plasticity and stress resilience.

Future research should investigate the long-term effects of TE activation and silencing under chronic stress and explore the potential adaptive significance of these processes in polar fish facing ongoing climate change.

## Materials and methods

### Experimental design

Adult individuals of *T. bernacchii* (Boulenger, 1902) (*n* = 12, average length = 23.76 ± 2.99 cm, average weight = 211.92 ± 80.40 g) were sampled at the end of October 2022, from the Ross Sea at Baia Terra Nova (74°42′S, 164°7′E) using hand lines with artificial baits at depths ranging from 60 to 100 m. After capture, the specimens were temporarily kept in buckets and then transported to the aquarium facility of the Mario Zucchelli Italian research station. There, they were housed in aerated 100 L-tanks with a continuous flow of seawater sampled from a depth of 5 m to replicate their native environmental conditions. Fish underwent a five-day acclimation period, during which the water temperature was kept constant at 0.01 ± 0.02 °C, as required by logistical limitations^78^. The tanks were also covered to reproduce the low-light conditions typical of their natural environment and to avoid stress induced by human presence. During acclimation, fish were not fed; water quality and temperature were monitored to ensure stable and optimal conditions. Recovery was additionally assessed by observing behavioral indicators.

Before the start of the experiment, fish were randomly and equally divided into two tanks, the control and the experimental group. Both were maintained for a total of 15 days in which the control group was set at a constant temperature of 0 °C while the experimental group was initially kept at a 0 °C for one day, followed by a gradual increase of + 1 ± 0.24 °C within 24 h and then maintained at this temperature for four days. Afterwards, the temperature was increased by a further degree and maintained for an additional four days, and finally this was repeated until + 3 ± 0.26 °C was reached over fifteen days. The temperature increase protocol was adapted from previous studies on Antarctic fish^79,80^. The gradual increase of + 1 °C every four days was implemented both to minimize mortality and to reflect natural thermal dynamics, while also accounting for logistical constraints inherent to Antarctic fieldwork. The selected temperature levels are ecologically relevant: +1 °C is slightly lower than the maximum summer temperature occasionally recorded at Baia Terra Nova (+ 1.5 °C), while + 3 °C is approximately twice this maximum, simulating an acute marine heatwave scenario. These values are consistent with temperature variations measured by multi-parameter probes deployed at 25 m depth in Terra Nova Bay.

At the end of the experiment fish were anesthetised using clove oil (diluted to 50 µL/L) to ensure minimal stress and human handling. Once unresponsive, fish were euthanised by severing the spinal cord following ethical guidelines for animal experimentation. For each fish, the heart was divided immediately after excision, and one portion was allocated for RNA extraction and treated with RNAlater^®^. The other portion was flash frozen in liquid nitrogen and stored at −80 °C served as backup sample.

Three samples were harvested on the fifth and fifteenth days to obtain biological replicates for both the control (CT5 and CT15) and the experimental group (T1 and T3). Sample collection and animal research methods complied with the Italian Ministry of Education, University and Research regulations concerning activities and environmental protection in Antarctica and with the Protocol on Environmental Protection to the Antarctic Treaty, Annex II, Art. 3. All the activities on animals performed during the XXXIII Italian Antarctic Expedition were supervised by a PNRA Ethics Referent, acting on behalf of the Italian Ministry of Foreign Affairs. In particular, the required data for the project identification code PNRA16_00099 are as follows. Name of the ethics committee or institutional review board: Italian Ministry of Foreign Affairs. Name of PNRA Ethics Referent: Dr. Carla Ubaldi, ENEA Antarctica, Technical Unit UTA. All experiments were performed under the U.K. Animals (Scientific Procedures) Act, 1986 and associated guidelines; EU Directive 2010/63/EU; and Italian DL 2014/26 for animal experiments. All methods are reported in accordance with ARRIVE guidelines.

### RNA isolation and sequencing

Total RNA extraction from liver and gill tissues was performed using TRIzol reagent (Thermo Fisher Scientific) following the manufacturer’s instructions. The quality of RNA samples was assessed by agarose gel electrophoresis. RNA samples were treated with DNase I (Thermo Fisher Scientific) and assayed for quantity using Qubit™2.0 (Thermo Fisher Scientific) and for quality with NanoDrop ™ 2000 (Thermo Fisher Scientific). The high-quality RNA biological replicates for tissues and conditions of interest were sent to IGA Tech (IGA Technology Services Srl, Udine, Italy) for sequencing. In all cases, RNA concentrations were > 1 µg/µl, with an RNA integrity number (RIN) > 6. Libraries for RNA-Seq were performed with Zymo-Seq RiboFree Total RNA library preparation kit (Zymo Research) producing paired-end 150 bp raw reads on NovaSeq 6000 (Illumina) platform. Sequencing data are available in NCBI under BioProject: PRJNA1345203.

### Transcriptional activity and differential expression of TEs

All the RNA-seq raw paired-end data from biological replicates of liver and gill tissues (Supplementary Table S3) were imported into CLC Genomics Workbench v.12 (Qiagen) and trimmed to remove low-quality bases/reads and sequencing adapters using default parameters. *De novo* transcriptomes were assembled using default parameters and then their completeness was assessed using BUSCO v.5.0.0^81^, with the Actinopterygii OrthoDB v.10 database as reference^82^.

In order to assess the transcriptional contribution of TEs, we identified TEs in the *de novo* assembled transcriptome with RepeatMasker v.4.1.0 (http://www.repeatmasker.org/cgi-bin/WEBRepeatMasker, accessed on 10 October 2024), employing a custom TE genome library of *T. bernacchii*, built as here briefly described. Firstly, the genome of *T. bernacchii* (accession number GCA_902827165.1) was downloaded from NCBI GenBank (https://www.ncbi.nlm.nih.gov/genome/) and a species-specific *de novo* TE library was constructed following the methods outlined in Carotti et al.^59^. RepeatScout v.1.0.6^83^ was employed to identify TEs and the resulting “build_lmer_table” was masked using RepeatMasker. Filtering steps were applied to exclude sequences repeated fewer than 10 times and to remove sequences not identified as TEs performing BLASTX^84^ searches against Uniprot-Swissprot database^85^ and InterProScan v5-34–73.0.0^86^ using a threshold e-value of 1 × 10^− 50^. The non-matching elements were further analyzed using HMMER^87^ to detect integrase, reverse transcriptase, and transposase domains (e-value < 1 × 10^− 5^). The remaining sequences (excluding simple tandem repeats) were classified using TEclass-2.13 (https://www.bioinformatics.uni-muenster.de/tools/teclass/index.hbi?, accessed on 13 January 2025). The resulting library was used to mask the *de novo* liver and gill transcriptomes assembled. For the transcribed TE sequences, values obtained from mapping of replicate trimmed reads against the transcriptome were used to calculate their overall contribution as a percentage of mapped reads for each sample (CT5, CT15, T1, T3) and tissue (liver and gills) considered in this study. The results were graphically represented in heatmaps using Rstudio^88^ packages (readxl^89^, ComplexHeatmap^90^, RColorBrewer^91^, magick^92^, and circlize^93^. Statistical analysis was performed in R using the vegan package^94^. A PERMANOVA (Permutational Multivariate Analysis of Variance) was conducted to assess differences in transposon expression patterns among experimental groups, based Euclidean distance matrices and 999 permutations; p-values < 0.05 were considered statistically significant.

For each tissue, to evaluate the differentially expressed TEs (DETEs), we used TEtranscripts v2.2.3^95^ between pairwise comparisons for each experimental vs. control group, according to the following scheme: T1 vs. CT5, T3 vs. CT15, CT15 vs. CT5, T3 vs. T1. Comparisons between gills vs. liver were performed as follow: gills CT5 vs. liver CT5, gills CT15 vs. liver CT15, gills T1 vs. liver T1, and gills T3 vs. liver T3. For this analysis, the input files included the BAM files of replicates sorted by position using SAMtools^96^, gene annotation file derived from NCBI (GCF_902827165.1), and TE annotation file generated from RepeatMasker output file. DETEs with Log2 Fold Change > |2| and the statistically significant threshold -Log10 (p-adj) = 0.05 were visualized in Rstudio^88^ using ggplot2^97^, dplyr^98^ and patchwork^99^ packages.

#### Transcriptional activity of genes involved in TEs silencing

Using TBLASTN^84^ genes of interest were searched and characterized in the RNA-seq data considered. The set of genes encoding proteins involved in TE controlling systems included: for *Argonaute* gene subfamily: *Argonaute RISC Component 1* (*ago1*), *Argonaute RISC Component 2* (*ago2*), *Argonaute RISC Component 3a* (*ago3a*), *Argonaute RISC Component 3b* (*ago3b*), *Argonaute RISC Component 4* (*ago4*); for heterochromatinization: *chromobox homolog 1a* (*cbx1a*), *chromobox homolog 1b* (*cbx1b*), *chromobox homolog 3a* (*cbx3a*), *chromobox homolog 3b* (*cbx3b*), *chromobox homolog 5* (*cbx5*), *DNA (cytosine-5-)-methyltransferase 1* (*dnmt1*), *DNA (cytosine-5-)-methyltransferase 3 alpha* (*dnmt3α*), *DNA (cytosine-5-)-methyltransferase 3beta* (*dnmt3β*) and, *SET domain bifurcated histone lysine methyltransferase 1b* (*setdb1b*); for NuRD complex *chromodomain helicase DNA binding protein 3* (*chd3*), *chromodomain helicase DNA binding protein 4a* (*chd4a*), *chromodomain helicase DNA binding protein 4b* (*chd4b*), *histone deacetylase 1b* (*hdac1b*), *methyl-CpG binding domain protein 2* (*mbd2*), *methyl-CpG binding domain protein 3a* (*mbd3a*), *methyl-CpG binding domain protein 3b* (*mbd3b*), *metastasis associated 1* (*mta1*), *metastasis associated 1 family*,* member 2* (*mta2*), *metastasis associated 1 family*,* member 3* (*mta3*), *GATA zinc finger domain containing 2Ab* (*gatad2ab*), *GATA zinc finger domain containing 2B* (*gatad2b*), *retinoblastoma binding protein 4* (*rbbp4*), *retinoblastoma binding protein 7* (*rbbp7*) and, *Tripartite Motif Containing 33* (*trim33*).

The transcriptional activity values of the considered genes were calculated as Transcript per Million (TPM) and were calculated using the pipeline described in our previous work^39^ using mapping parameters: length fraction = 0.9 and similarity fraction = 0.9. Logarithmic TPM values were graphically represented in heatmaps using Rstudio^88^ packages (readxl^89^, ComplexHeatmap^90^, RColorBrewer^91^, magick^92^, and circlize^93^. Statistical analysis was performed in R using the vegan package^94^. A PERMANOVA (Permutational Multivariate Analysis of Variance) was conducted to assess differences in gene expression patterns among experimental groups, based on Euclidean distance matrices and 999 permutations; p-values < 0.05 were considered statistically significant.

##### Kimura distance-based TE age distribution in the genome and transcriptomes of *T. bernacchii*

The Kimura distance landscapes, representing rates of transitions and transversions, were generated using the “calcDivergenceFromAlign.pl” and “createRepeatLandscape.pl” scripts provided by the RepeatMasker package. This analysis was conducted at the genome level and for expressed TEs identified in the assembled transcriptomes for both liver and gills at control and test conditions (CT5, CT15, T1, T3). This approach was also applied to DETEs obtained using TEtranscripts.

## Supplementary Information

Below is the link to the electronic supplementary material.

Supplementary Material 1

## Data Availability

RNA-seq data analysed during this study were deposited in the Sequence Read Archive (SRA) under the accession numbers reported in supplementary table S4.
