# Supplementary material for: Transcriptional response of transposable elements to thermal stress in the Antarctic fish Trematomus bernacchii
Source: Sci Rep. 2026 Jan 7;16:3440. doi: 10.1038/s41598-025-33487-5 (PMC12834978; doi:10.1038/s41598-025-33487-5)
Supplement: Supplementary file 1 — Supplementary Material 1 [file 41598_2025_33487_MOESM1_ESM.pdf]

Supplementary Material

Transcriptional response of transposable elements to thermal stress in the Antarctic fish *Trematomus bernacchii*

Edith Tittarelli[†1,2], **Elisa Carotti**[†1], **Claudia Palladinelli**[1], **Marco Barucca**[1], **Federica Carducci**[1], **Gianfranco Santovito**[3], **Elisabetta Piva**[3], **Adriana Canapa**[1], **Maria Assunta Biscotti**[1]*

1 Dipartimento di Scienze della Vita e dell'Ambiente, Università Politecnica delle Marche, Via Brecce Bianche, 60131, Ancona (Italy).

2 Scuola Universitaria Superiore Pavia – IUSS, Piazza della Vittoria n.15, 27100, Pavia (Italy).

3 Dipartimento di Biologia, Università di Padova, Viale Giuseppe Colombo 3, 35131, Padova (Italy).

[*]Corresponding author e-mail: m.a.biscotti@univpm.it

[†] These authors equally contributed.

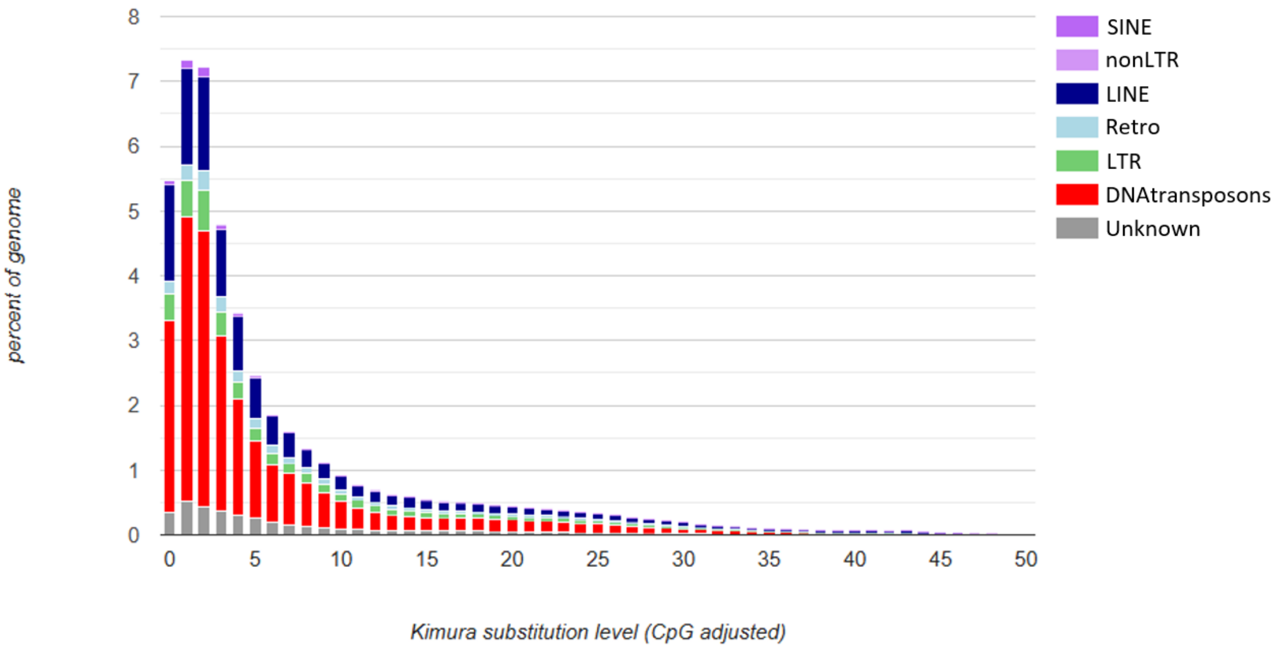

Supplementary Figure S1. Supplementary Figure S1. Repeat landscape plot obtained by Kimura distance-based copy divergence analyses of TEs in *T. bernacchii* genome.

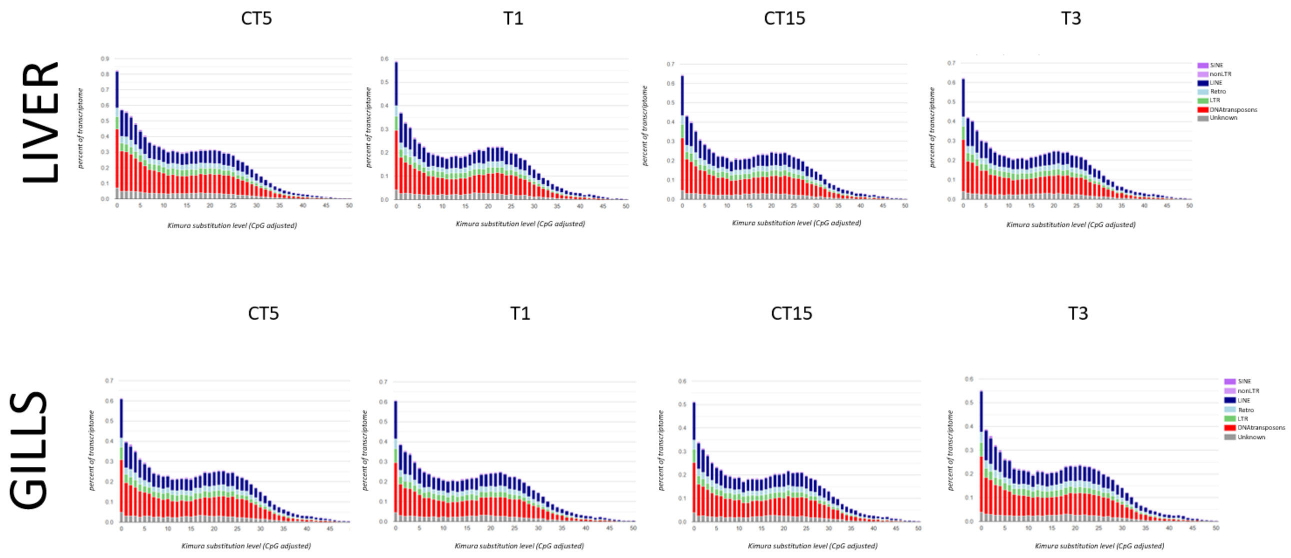

Supplementary Figure S2. Repeat landscape plots obtained by Kimura distance-based copy divergence analyses of TEs in *T. bernacchii* liver and gill transcriptomes.

```
==================================================
file name: GCF_902827165.1_fTreBer1.1_genomic.fna
sequences:           864
total length:  867125071 bp  (867007417 bp excl N/X-runs)
GC level:         40.94 %
bases masked:  381670751 bp ( 44.02 %)
==================================================
               number of      length   percentage
               elements*    occupied  of sequence
--------------------------------------------------
Retroelements       513116    121121702 bp   13.97 %
   SINEs:            20812      3190730 bp    0.37 %
   Penelope              0            0 bp    0.00 %
   LINEs:           336755     84729814 bp    9.77 %
    CRE/SLACS            0            0 bp    0.00 %
     L2/CR1/Rex          0            0 bp    0.00 %
     R1/LOA/Jockey       0            0 bp    0.00 %
     R2/R4/NeSL          0            0 bp    0.00 %
     RTE/Bov-B           0            0 bp    0.00 %
     L1/CIN4             0            0 bp    0.00 %
   LTR elements:    155549     33201158 bp    3.83 %
     BEL/Pao             0            0 bp    0.00 %
     Ty1/Copia           0            0 bp    0.00 %
     Gypsy/DIRS1         0            0 bp    0.00 %
       Retroviral        0            0 bp    0.00 %

DNA transposons     615799    188452144 bp   21.73 %
   hobo-Activator        0            0 bp    0.00 %
   Tc1-IS630-Pogo        0            0 bp    0.00 %
   En-Spm                0            0 bp    0.00 %
   MuDR-IS905            0            0 bp    0.00 %
   PiggyBac              0            0 bp    0.00 %
   Tourist/Harbinger     0            0 bp    0.00 %
   Other (Mirage,        0            0 bp    0.00 %
    P-element, Transib)

Rolling-circles          0            0 bp    0.00 %

Unclassified:       282155     50355077 bp    5.81 %

Total interspersed repeats:   359928923 bp   41.51 %


Small RNA:               0            0 bp    0.00 %

Satellites:              0            0 bp    0.00 %
Simple repeats:     260980     20344954 bp    2.35 %
Low complexity:      25391      1396874 bp    0.16 %
==================================================

* most repeats fragmented by insertions or deletions
  have been counted as one element
```

Supplementary Table S1. Output of RepeatMasker analyses on *T. bernacchii* genome.

Tissue	Comparisons	Expression	DETEs	DNA transposons	LINE	LTR	non-LTR	Retro	SINE	Unknown	Total DETEs
Liver	CT15 vs CT5	Up	2	—	—	1	—	1	—	—	75
		Down	73	19	21	10	1	10	1	11	
		% of DETEs		25	28	15	1	15	1	15	
	T1 vs CT5	Up	5	1		1		1		2	365
		Down	360	86	124	64	1	38	4	43	
		% of DETEs		24	34	18	0	11	1	12	
	T3 vs CT15	Up	—	—	—	—	—	—	—	—	0
		Down	—	—	—	—	—	—	—	—	
		% of DETEs		0	0	0	0	0	0	0	
	T3 vs T1	Up	3	—	2	—	—	1	—	—	5
		Down	2	—	2	—	—	—	—	—	
		% of DETEs		0	80	0	0	20	0	0	
Gills	CT15 vs CT5	Up	10	1	3	3	—	—	—	3	18
		Down	8	3	3	2	—	—	—	—	
		% of DETEs		22	33	28	0	0	0	17	
	T1 vs CT5	Up	68	8	26	15	—	13	1	5	86
		Down	18	6	10	2	—	—	—	—	
		% of DETEs		16	42	20	0	15	1	6	
	T3 vs CT15	Up	122	23	48	24	2	16	2	7	138
		Down	16	3	5	2	—	4	—	2	
		% of DETEs		19	38	19	1	14	1	7	
	T3 vs T1	Up	15	1	6	3	—	4	—	1	27
		Down	12	3	4	2	—	2	—	1	
		% of DETEs		15	37	19	0	22	0	7	

Comparisons	Time point	Expression	DETEs	DNA transposons	LINE	LTR	non-LTR	Retro	SINE	Unknown	Total DETEs
Gills vs liver	CT5	Up	200	50	62	28	3	37	2	18	2339
		Down	2139	511	707	401	14	305	32	169	
		% of DETEs		24	33	18	1	15	1	8	
	CT15	Up	320	63	113	54	3	55	3	29	1386
		Down	1066	259	339	205	5	159	21	78	
		% of DETEs		23	33	19	1	15	2	8	
	T1	Up	421	91	142	73	7	68	6	34	9663
		Down	542	134	191	102	3	65	8	39	
		% of DETEs		23	35	18	1	14	1	8	
	T3	Up	509	121	166	81	6	79	10	46	1215
		Down	706	171	246	124	3	104	13	45	
		% of DETEs		24	34	17	1	15	2	7	

Supplementary Table S2. Count table of up and down DETEs.

	CT5	CT15	T1	T3
Gills	SRR35788766	SRR35788763	SRR35788760	SRR35788756
	SRR35788765	SRR35788762	SRR35788759	SRR35788755
	SRR35788764	SRR35788761	SRR35788757	SRR35788754
	**CT5**	**CT15**	**T1**	**T3**
Liver	SRR35788770	SRR35788753	SRR35788750	SRR35788747
	SRR35788769	SRR35788752	SRR35788749	SRR35788768
	SRR35788758	SRR35788751	SRR35788748	SRR35788767

Supplementary Table S3. Accession numbers of RNAseq raw data obtained from gills and liver of *T. bernacchii* related to control and exposure conditions.
